# Active Site Detection by Spatial Conformity and Electrostatic Analysis—Unravelling a Proteolytic Function in Shrimp Alkaline Phosphatase

**DOI:** 10.1371/journal.pone.0028470

**Published:** 2011-12-08

**Authors:** Sandeep Chakraborty, Renu Minda, Lipika Salaye, Swapan K. Bhattacharjee, Basuthkar J. Rao

**Affiliations:** Department of Biological Sciences, Tata Institute of Fundamental Research, Mumbai, India,; University of South Florida College of Medicine, United States of America

## Abstract

Computational methods are increasingly gaining importance as an aid in identifying active sites. Mostly these methods tend to have structural information that supplement sequence conservation based analyses. Development of tools that compute electrostatic potentials has further improved our ability to better characterize the active site residues in proteins. We have described a computational methodology for detecting active sites based on structural and electrostatic conformity - **C** *ata* **L** *yti*c **A** *ctiv*e **S** *it*e **P** *redictio*n (CLASP). In our pipelined model, physical 3D signature of any particular enzymatic function as defined by its active sites is used to obtain spatially congruent matches. While previous work has revealed that catalytic residues have large *pKa* deviations from standard values, we show that for a given enzymatic activity, electrostatic potential difference (PD) between analogous residue pairs in an active site taken from different proteins of the same family are similar. False positives in spatially congruent matches are further pruned by PD analysis where cognate pairs with large deviations are rejected. We first present the results of active site prediction by CLASP for two enzymatic activities - β-lactamases and serine proteases, two of the most extensively investigated enzymes. The results of CLASP analysis on motifs extracted from Catalytic Site Atlas (CSA) are also presented in order to demonstrate its ability to accurately classify any protein, putative or otherwise, with known structure. The source code and database is made available at www.sanchak.com/clasp/. Subsequently, we probed alkaline phosphatases (AP), one of the well known promiscuous enzymes, for additional activities. Such a search has led us to predict a hitherto unknown function of shrimp alkaline phosphatase (SAP), where the protein acts as a protease. Finally, we present experimental evidence of the prediction by CLASP by showing that SAP indeed has protease activity *in vitro*.

## Introduction

The PDB database has more than 60,000 protein structures to date [1]. Classification of proteins is the logical outcome of the motivation to add a sense of order in this rapidly growing database [2]–[7]. Identifying the catalytic sites in a protein is an important first step towards characterizing its function. Active site determination by site directed mutagenesis methods is often time consuming and labour intensive to apply on the large number of new proteins being discovered on a continual basis.

Currently several methods are available for identifying active site residues in a protein, amongst which methods based on electrostatic potentials are increasingly gaining ground. Electrostatic interactions determine various properties of biomolecules such as catalytic activity, ligand binding, structure and stability [8]. Finite difference Poisson-Boltzmann (FDPB) electrostatics is used to compute potential differences (PD) and *pKa* values from charge interactions in proteins [9]–[11]. This continuum model of charges led to the development of tools for studying electrostatic interactions [12]–[14]. These tools implement fast numerical approximation of solutions to the linearized Poisson-Boltzmann equation [15]. Active site residues have *pKa* values that differ considerably from their intrinsic values [16], [17]. These large *pKa* deviations from standard values have been used for predicting active site residues [18]–[20]. The *pKa* perturbations of the ionizable residues on the surface of proteins are minimal, while those buried in the interior of the protein show considerable shifts [21], [22]. Significant shifts in *pKa* values usually stem from interactions with other ionizable residues, hydrogen bonding with non-ionizable residues and its exposure to the bulk solvent. The buried nature of residues in active sites often results in their high electrostatic potential which has been used as a differentiator between catalytic and other residues [23], [24].

There are essentially two flavors in the existing models of active site prediction. The first one requires a motif of a known enzymatic function to search for the same within a protein of interest. Several web-servers give access to 3D motif based search methods in proteins with known structures. SPASM [25] and RASMOT [26] search for a specified motif in the queried protein. MultiBind recognizes 3D-binding patterns common to several protein structures against precomputed ligands [27]. PAR-3D uses 3D motifs to identify several different functions but is restricted to a few protein activities [28]. Superimpose allows searching for a specific 3D motif in protein structure databases [29]. A method that detects recurring three-dimensional side-chain patterns of amino acids in proteins has concluded that alkaline phosphatases and aminopeptidases have evolved convergently [30]. A recent method, ProBis, detects structurally similar sites on protein surfaces by a local structural alignment technique [31], and compares its results with other analogous methods [32]–[34]. Though most of these methods do a spatial match of motifs, they do not factor in properties related to electrostatic potentials for eliminating false positive matches. Our current method addresses this shortcoming.

The other approaches typically describe learning from known active sites across a variety of catalytic functions and use these learnt properties to predict active sites. Finally these results must be tested either *in silico*, *in vitro* or *in vivo* in order to validate the assigned putative activity. The search for active sites in these *in silico* methods are generally guided by time tested thumb rules [35]. Conservation of residues in a family of proteins, their presence in a cleft, solvent accessibility and hydrophilicity are a few such properties. RESBOOST integrates the characteristics of catalytic residues and presents them as a logical expression of simple rules [36]. DISCERN combines phylogenetic scores, information from structure, properties computed for structural neighbours and a statistical regularization to control for overfitting [37]. Another method also combines multiple predictors into a single classifier [38]. ConCavity uses evolutionary sequence conservation estimates with structure-based surface pocket prediction [39]. Other methods that analyze evolutionary conservation essentially rely on clusters of conserved sites [40], or create databases of sequence segments that encode structural attributes of the binding pockets [41]. However none of the above mentioned methods use electrostatic features in the search process, while a few do not provide much flexibility in modulating the specificity and sensitivity.

Some methods developed recently use electrostatic properties to discriminate active sites. Active sites are known to occur in electrostatically unfavourable environments [18], [42]. Often active sites involve ionizable groups where anomalous *pKa* values have been tagged with catalytic activity [19]. Q-SiteFinder does ligand binding site prediction by computing energetically favourable binding sites on the surface of a protein [43]. THEMATICS reports that residues involved in catalysis can be differentiated from ordinary residues based on chemical properties [20]. POOL uses electrostatic features from THEMATICS and combines geometric properties to estimate probabilities that specific residues belong to an active site and assigns a likelihood ranking of all ionizable residues in a given protein [44]. Another innovative method [45] tries to dock high-energy intermediates of various metabolites listed in a database [46] and successfully predicts the function of an unknown protein.

**Figure 1:**
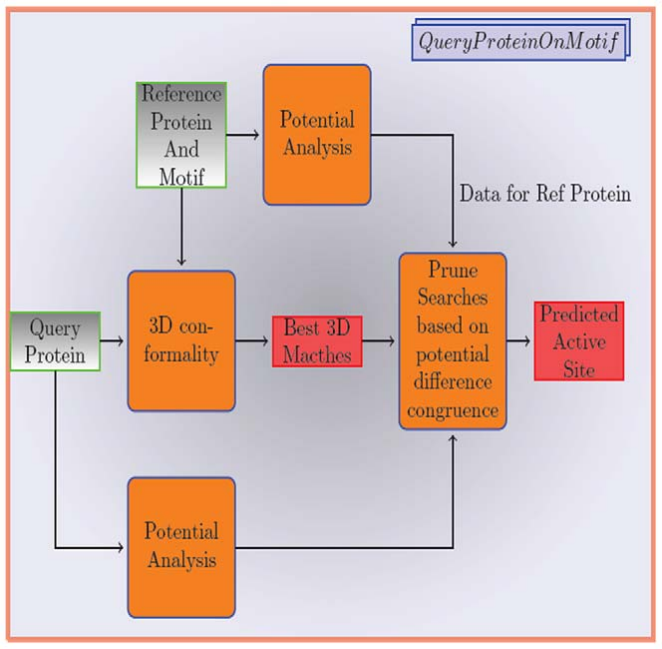
CLASP flow. The flow for using a motif from a protein with known function and 3D structure in identifying active sites in an unknown protein. Electrostatic potentials are precomputed for proteins already in the PDB Database or computed on the fly for new proteins. These properties are then used to prune out false positives.

Catalysis often involves movement of charges across specific pairs of residues along paths that are spatially well defined. Therefore, active site predictions must invoke methods that combine analysis of electrostatic potentials with those of spatial congruence. Our current method precisely does this (Fig. 1). In this work we show a strong correlation in the electrostatic PD between sets of cognate residues in active sites. For a given enzymatic activity, pairs of residues in an active site taken from various proteins of the same family show high correlation of electrostatic PD with small standard deviation. In other words these PD lie in a narrow band. We exploit this correlation to prune out false matches from spatial congruence matches. Therefore, PD analysis adds an additional filter for improving accuracy in active site prediction. Moreover, electrostatic analyses implicitly takes into account macroscopic features such as substrate accessibility, cleft geometry, etc that are critically involved in defining an active site.

We present here a computational methodology, **C** *ata* **L** *yti*c **A** *ctiv*e **S** *it*e **P** *redictio*n (CLASP), for active site detection. A motif from a protein of known function and 3D structure provides the initial template for searching spatially congruent matches. These matches are then pruned by the observation that the PD in pairs of residues in active sites is highly correlated. Our method can interrogate any given hypothetical protein with known structure for motifs derived from the Catalytic Site Atlas (CSA) [35] database and rank the matches. This further allows CLASP in classifying promiscuous activities associated with the protein (Figure S1). Any method to classify proteins or predict catalytic residues is subject to an inherent trade-off between sensitivity and specificity. CLASP provides precise control to obtain acceptable levels of sensitivity and specificity by enabling the choice of stereochemically equivalent residues. Alkaline phosphatases (AP) are one of the widely studied promiscuous proteins. We probed APs for various motifs from CSA using CLASP. Such an analysis uncovered a uniquely promiscuous proteolytic function in shrimp alkaline phosphatase (SAP). The predicted protease active site overlaps with the active site of AP. We substantiate this prediction by providing experimental evidence *in vitro*: we have demonstrated an additional protease activity of SAP, acting not only on a substrate *in trans* but also on itself and that its AP activity is inhibited by trypsin inhibitors. Interestingly, this inhibition has been shown in other APs as well.

## Results

### Active Site Prediction

We first present the results of active site prediction by CLASP for two enzymatic activities - β-lactamases and serine proteases, two of the most extensively investigated enzymes. Subsequently, the results of CLASP analysis is presented on motifs extracted from CSA that demonstrates its ability to accurately classify any protein, putative or otherwise, with known structure. Finally, we present *in vitro* evidence of the prediction by CLASP that SAP has protease activity.

#### β-lactamases

β-lactamases are the chief cause of bacterial resistance to penicillin, cephalosporin and related β-lactam compounds. Several well conserved motifs in β-lactamases have been outlined in detail which reveals the amino acid residues critically involved in catalysis [47]. We chose the catalytically involved residues {Ser70, Lys73, Ser130, Lys234} to represent the Class A, C and D β-lactamases (Table S1).

Adaptive Poisson-Boltzmann Solver (APBS) was used to compute the electrostatic PD between pairs of residues in this motif [14]. The values of the PD between residue pairs have high correlation as evidenced by small standard deviation (Table 1 and Table S2), implying chemical crosstalk between them. Although correlated, certain residue pairs are charge neutral {Ser70, Ser130} and therefore may not be functionally relevant as a pair. A study which elaborates the acylation mechanism of β-lactamases at 0.88 Å shows that the residue pairs with high PD and correlation indeed crosstalk with each other, while the ones with low PDs do not [48]. The *pKa* of Asp66 in hen egg white lysozyme (PDB id: 2LZT) calculated from the PD obtained using APBS concurs with the reported values [49], thereby validating the accuracy of our PD estimations. Moreover the *pKa* of Lys73 in a β-lactamase (PDB id: 2G2U) calculated by APBS matched well with those values reported earlier by 3 different methods of *pKa* computation [50].

**Table 1:** Adaptive Poisson-Boltzmann Solver (APBS) calculated potential differences in residue pairs in β-lactamases using a Class A β-lactamase motif.

PDB id	Class	S70/K73OG/NZ	S70/S130OG/OG	S70/R234OG/NH1	K73/S130NZ/OG	K73/R234NZ/NH1	S130/R234OG/NH1
2G2U	A	−201.7	22.3	−250.3	224.0	−48.6	−272.7
2QZ6	C	−196.5	15.9	−240.3	212.4	−43.7	−256.1
2HP5	D	−215.1	−12.8	−228.3	202.3	−13.2	−215.5
Mean		−204.4	8.5	−239.6	212.9	−35.2	−248.1
SD		7.8	15.3	9.0	8.9	15.7	24.0

Potential differences are in dimensionless units of kT/e (k is Boltzmann's constant, T is the temperature in K and e is the charge of an electron).

**Figure 2:**
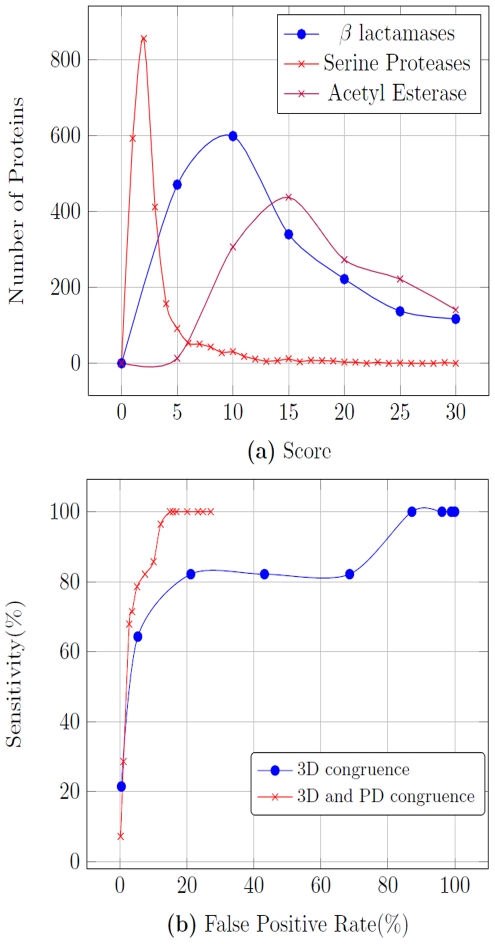
CLASP scores. (a) CLASP scores on a set of ∼3,000 non-redundant proteins for serine proteases, β-lactamases and acetyl esterase having 3, 4 and 5 residues in the active site motif. (b) Sensitivity versus FPR curves for running CLASP on a set of ∼500 proteins for a β-lactamase motif. The 500 proteins that were the best candidates using a 3D match on all the ∼50,000 proteins in the PDB database, hence our results will be better on the whole set of proteins.

The CLASP scores obtained on a list of ∼3000 non-redundant proteins (sequence-dissimilar PDB polypeptide chains, http://www.ncbi.nlm.nih.gov/Structure/VAST/nrpdb.html) shows its discriminating power (Fig. 2a). Lower scores denote lesser deviation and consequently a better match. It is evident from the analysis, more the number of specified residues in the active site motif, lesser is the likelihood of scoring a non-related protein (false positive). The rightward shift of the distribution of scores obtained for the serine protease motif vis-a-vis that of β-lactamases also highlights this point since the former motif has lesser (3) residues for analysis. Expectedly, β-lactamases and related proteins like penicillin binding proteins (PBP) and transpeptidases score the best with β-lactamase motif (Table 2). Surprisingly, phospholipase C also shows up as related protein with better score than a salt-tolerant glutaminase (PDB id: 3IHB) that has been recently shown to be related to β-lactamases [51]. The strong correlation in pairs of residues in the active site as seen in β-lactamases is also found in PBPs, which are known to interact with β-lactam antibiotics (Table S2). We recorded a significant decline in the false positives (80% to 20%) when we combined PD correlation to that of 3D congruence, thereby enhancing the discrimination (Figure S2). This was measured for a set of proteins that were scored as the top 500 hits in a 3D match on all the ∼50,000 proteins in the PDB database. An example of a protein that is pruned out as a false positive is a calcium binding lysozyme (PDB id: 2EQL). In this protein 3D congruence had revealed that the predicted active site residues {Ser51/Lys62/Asn60/Lys46} showed a maximum pair wise distance deviation of less than 1 Å, when compared to the β-lactamase motif, thereby scoring this protein as a possible β-lactamase. However, the PD between the same residue pairs did not correlate well, suggesting that it is unlikely to be a β-lactamase (pair wise deviation in the PDs are {9,229,122,219,113,106} in units of kT/e, while the pair wise deviations in distances are {0.2,0.7,0.5,0.3,0.7,1} in Å.

**Table 2:** Best matches on the set of ∼3,000 non-redundant proteins using a β-lactamase motif.

PDB	Annotation	Score
1JTG	β-lactamase TEM	0.7
3HUM	penicillin-binding protein	0.9
3DW0	Class A β-lactamase carbapenemase KPC-2	1.2
1SKF	transpeptidase	2.5
1QME	penicillin-binding protein	2.9
1FOF	β-lactamase oxa-10	3.2
1DJH	phospholipase C	4.5
3IHB	salt-tolerant glutaminase	5.2

CLASP provides the ability to constrain the search by selecting the groups of stereochemically equivalent residues (Table S1). The predicted residues (Table S3) show the Tyr150 in Class C β-lactamases as part of the active site in place of the stereochemically equivalent Ser130 in Class A. Since CLASP has the flexibility of choosing residues within a chemically equivalent group (e.g. Ser group), Tyr150 is identified as the third residue which the methods used by CSA could not predict. It is to be noted that for some Class C β-lactamases, a different Tyr residue (Tyr112 in PDB id: 2QZ6) has a better match than Tyr150. While this might be a false match, it is also quite possible that this residue is actually critical for catalysis. Table S3 also highlights the fact that the scoring definitely ranks Class A enzymes first since we have used a Class A β-lactamase motif. A Class C β-lactamase (PDB id: 3GQZ) is an exception and scores in the range of Class A β-lactamases, possibly due to the fact that this enzyme has had conformational changes due to fragment binding [52].

#### Serine Proteases

Serine proteases are grouped based on structural homology and are then further subgrouped into families with similar sequences [53], [54]. The two major families, chymotrypsin and subtilisin, are a classical example of convergent evolution where the catalytic Ser-His-Asp triad shows virtually similar geometry in the structurally different chymotrypsin and subtilisin [55]. The motif from trypsin – {Ser195, His57, Asp102} -was chosen for representing serine proteases. All residue pairs show high correlation in serine proteases in their PD (Table 3a).

**Table 3:** Pairwise distances and potential differences in the catalytic triad in serine proteases (a) and in predicted active sites in alkaline phosphatases (S/D, H/D, S/H) (b).

		Distances			Potentials		
Serine Proteases							
a	1A0J (Trypsin)	7.8	5.5	3.3	−144.1	−123.4	104.8
	1OS8 (Trypsin)	7.5	5.5	2.8	−139.5	−124.8	168.3
	1BH6 (Subtilisin)	6.5	4.9	4.1	−90.5	−113.0	122.4
	1GCI (Subtilisin)	5.6	4.55	4.7	−109.5	−108.0	69.6
	Alkaline Phosphatases						
b	2X98(HSAP)	4.9	3.1	4.7	−131.0	−151.3	84.1
	1ALK(ECAP)	7.1	3.6	5.4	−210.0	−191.6	99.4
	1K7H(SAP)	7.7	3.2	4.6	−139.0	−139.8	137.5
	1EW2(PLAP)	5.0	3.7	5.1	−142.0	−16.4	139.7
	2IUC(TAP)	5.2	3.3	4.7	−136.0	−155.2	170.5
	3E2D(VAP)	7.0	3.8	5.0	−91.0	−115.4	116.9

Distances are in Å, potential differences are in dimensionless units of kT/e (k is Boltzmann's constant, T is the temperature in K and e is the charge of an electron).

#### Classification of proteins with known and unknown catalytic activities based on CSA Motifs

CSA provides catalytic residue annotation for enzymes in the PDB and is available online [35]. The database consists of an original hand-annotated set extracted from the primary literature and a homologous set inferred by PSI-BLAST [56]. The CLASP score is always zero when we probe the motif in the reference protein itself, i.e. when the template and the query are the same protein. We expect the CLASP score to be low when we probe this motif in another protein from the same family because of high spatial and PD congruence.

CLASP scores for enzymatic activities represented by 3, 4 and 5 active site residues are presented in Table 4. A set of specific proteins (left column) are queried for a given active site motif (top row) in its entire structure. Such a bioinformatic scan is expected to reveal the best spatial and electrostatic match as the least CLASP score. As expected, the querying motif is detected as the best match in the protein chosen from its cognate family in most cases. For example, a dehydratase motif scores best in a known dehydratase (PDB id: 1JCT). Since we have ordered the enzymes and the motifs similarly, the best matches should fall diagonally in the table. The analysis indeed reveals that it is so for 5 residue motifs without any exception. However for 4 residue motifs, pepsin (PDB id: 1AM5) is an exception that matches slightly better with a de-aminase motif than with itself. On the other hand, 3 residue motifs show larger number of false positives, such as hexokinase and chitinase matching well with each other. Most surprisingly CLASP matches amino-transferase motif significantly better with hexokinase than with itself (PDB id: 1IVR). We believe that CLASP analysis picks up larger false positives in 3 residue motifs. However, unless validated *in vivo* any low scoring match cannot be discarded as false based on the existing knowledge about that protein. For instance, a hypothetical protein (PDB id: 1P1M) matches best with a deaminase motif in CLASP analysis, which a recent study has predicted and shown to be a deaminase [45]. CLASP was able to predict catalytically active residues in this protein (His55,Glu203,Asp279,Asp113) on the basis of its close match (Table S4) with deaminase. This underscores the predictive power of CLASP.

**Table 4:** CLASP scores for enzymatic activities which have 3, 4 and 5 residues specified in their active sites.

		Hexokinase-2	aminotransferase	protease	chitinase
		1IG8	1ECX	1A0J	1CTN
3 residues	2NZT (Hexokinase-2)	1.3	3.0	4.6	1.3
	1IVR (aminotransferase)	0.4	4.0	5.8	3.6
	2TBS (protease)	2.1	5.3	0.4	3.9
	1RD6 (chitinase)	0.8	4.2	8.0	1.7

		deaminase	β-lactamase	lysozyme	pepsin
		1CD5	2G2U	1OBA	1MPP
4 residues	1P1M (deaminase)	4.3	10.9	5.4	9.4
	1JTG (β-lactamase)	11.3	0.7	3.8	11.1
	3H12 (lysozyme)	6.0	8.2	5.1	7.7
	1AM5 (pepsin)	5.0	12.0	6.1	6.0

		cutinase	dehydrogenase	dipeptidase	dehydratase
		1AGY	1J49	1FY2	1EC9
5 residues	1CUJ (cutinase)	7.4	16.8	16.2	17.0
	1F0X (dehydratase)	16.0	5.6	10.8	6.8
	2RAG (dipeptidase)	9.5	7.0	5.8	9.6
	1JCT (dehydratase)	10.7	7.1	12.8	3.2

The reference protein in which the motif is chosen from (top row) and the query protein (left column) belong to the same family. Ideally, the diagonal scores should be the lowest.

#### Performance measurement and comparison to existing methods

We evaluated the results using the measures of sensitivity, specificity and false positive rate (FPR), which are defined as:
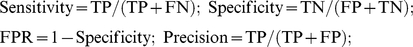
(TP = true positives, TN = true negatives, FP = false positives, FN = false negatives). Recall is identical to sensitivity. Ideally, sensitivity and FPR should be 1 and 0 respectively. As an example, one of the recent studies (RESBOOST) reported 90% sensitivity for a 9.8% FPR and 73% sensitivity for a 5.7% FPR [36]. Another method (DISCERN) reports 69% sensitivity at 18% precision [37]. The sensitivity versus FPR curves of CLASP results on a set of ∼500 proteins for a β-lactamase motif is presented in Fig. 2b, which shows a 86% sensitivity for a 10% FPR. In contrast, the same set with analysis employing 3D congruence alone achieves reduced sensitivity (70%) for the same FPR (10%), which reinforces the contribution of PD filtering in improving discrimination. The true positives (β-lactamases) have been selected based on their PDB annotation. It is relevant to state that the analysis pertains to a prefiltered set of 500 proteins which were obtained following a 3D match for β-lactamases for all the ∼50,000 proteins in the PDB database. Hence, our reported results are an underestimate and the true sensitivity on an unbiased whole set will be much higher.

#### Promiscuous proteolytic function in SAP

Based on this predictive power, we queried a well known promiscuous protein family, alkaline phosphatase (AP), using all motifs from the CSA database. We found that the 3 residue serine protease motif scores significantly well (Table 5a), thereby suggesting that AP may indeed have protease activity. We describe below *in vitro* evidence of the prediction made by CLASP.

**Table 5:** (a) CLASP scores in SAP (PDB id: 1K7H) using about 400 motifs in CSA (b) CLASP scores in the AP superfamily motif using a serine protease motif.

	PDB	Annotation	Score
a	2ISD	phospholipase C	1.087
	2AMG	maltotetrahydrolase	1.120
	2GSA	aminotransferase	1.264
	1CNS	chitinase	1.316
	1JS4	endo/exocellulase E4	1.329
	1EYI	fructose-1,6-bisphosphatase	1.381
	2OAT	aminotransferase	1.500
	1A0J	trypsin	2.084
	1JKM	esterase	2.372
	1AZW	iminopeptidase	2.606
	1ORD	decarboxylase	2.688
	1ESC	esterase	2.940
	2LPR	alpha-lytic protease	2.958
b	2GSN	pyrophosphatase(NPPs)	1.735
	1K7H	alkaline phosphatase	2.084
	1HDH	arylsulfatase	5.42
	1EJJ	phosphoglycerate mutase	5.46
	2VQR	phosphonate monoester hydrolase	5.620

**Figure 3:**
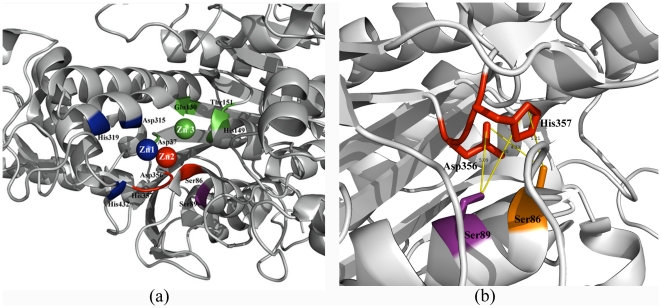
Active site in SAP. (a) The active site for SAP (PDB id: 1K7H) consists of 3 metal binding sites -M1 (Asp315,His319,His432) in blue binds a zinc, M2 (Asp37,Ser86,Asp356,His357) in red binds another zinc, while M3 (Asp37,His149,Thr151,Glu130) in green binds either a zinc or a magnesium ion. CLASP predicted active site residues for protease activity (Ser86,Asp356,His357) coincides with the M2 site containing serine. Another serine (Ser89), shown in purple, shows up as an alternative to Ser86 in the protease motif in CLASP analysis. (b) The protease activity in SAP might be ascribed to either of the two serines -Ser86 and Ser89. The distance of these serines from Asp356 and His357 is also shown.

**Figure 4:**
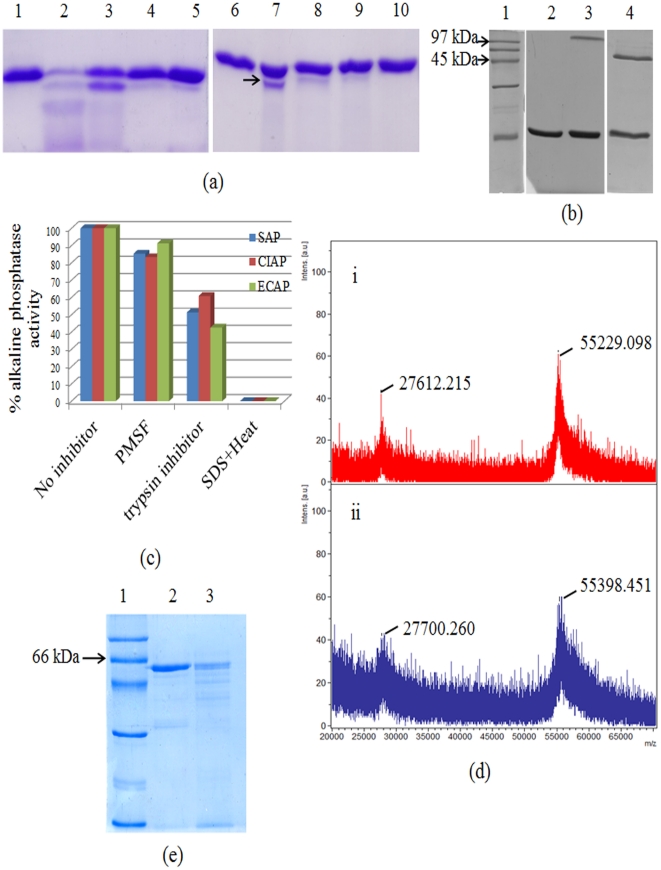
Experimental results. (a) Protease activity of SAP and the effect of different protease inhibitors. Substrate protein (UVI31+; lanes 1 & 6) was incubated with either Trypsin (lanes 2–5) or SAP (lanes 7–10) for over night at 37°C. Lanes 3–5 and 8–10 shows the effect of different protease inhibitors on the protease activity of Trypsin and SAP, respectively. The enzyme was pre-incubated with trypsin inhibitor (lane 3 & 8), PMSF (lanes 4 & 9) and SDS plus heat for 5 min before substrate addition (lanes 5 and 10). (b) Protease activity of CIAP and ECAP. Substrate protein (UVI31+; lane 2) was incubated with either CIAP (lanes 3) or ECAP (lane 4) overnight at 37°C, followed by sample analysis on a 15% SDS-PAGE. Lane 1, molecular weight marker. (c) Effect of different protease inhibitors on the phosphatase activity of SAP, CIAP and ECAP. Readings are a mean of three independent experiments. The AP activity of SAP, CIAP and ECAP in the absence of inhibitor have been normalized to 100%. (d) MALDI-mass spectrometry of (i) Native SAP and (ii) AEBSF-SAP complex. SAP was pre-incubated with AEBSF for 30 mins at room temperature. The reaction was stopped by adding 0.5% Trifluoroacetic acid. (e) Effect of EDTA, a metal ion chelator on the autolysis activity of SAP. SAP was incubated with (lane 3) or without (lane 2) EDTA (10 mM) for overnight at 37°C followed by analysis on a 12% SDS-PAGE. Lane 1, molecular weight marker.

Alkaline phosphatases (AP, EC 3.1.3.1) are homodimeric metalloenzymes that act through a phosphoseryl intermediate to produce free inorganic phosphate or transfer the phosphoryl group to other alcohols [57]–[59]. This class of enzymes have a broad range of substrate specificity which include proteins, DNA and small organic molecules. It is known that AP family enzymes have active sites with a high degree of promiscuity. CLASP predicted that AP (PDB id: 1K7H) will exhibit promiscuous protease activity at the metal binding site M2 (Fig. 3a). The spatial and electrostatic congruence of this motif in several APs is compared with that in serine proteases (Table 3). We tested this prediction by performing a protease assay *in vitro* on commercially available APs -shrimp alkaline phosphatase (SAP), *E. coli* alkaline phosphatase (ECAP) and calf intestinal alkaline phosphatase (CIAP). Trypsin (0.73 µM) digested the entire substrate protein (Fig. 4a, lane 2), while under similar reaction conditions SAP cleaved a measurable fraction (22.5% as measured by band quantification using Image J) of the substrate (lane 7). However, there was no detectable protease activity in CIAP and ECAP (Fig. 4b) (see discussion). Known inhibitors of serine proteases such as Phenylmethylsulfonyl fluoride (PMSF) and trypsin inhibitor inhibited the protease activity of both trypsin and SAP (Fig. 4a, lanes 3–4 and lanes 8–9, respectively). SDS treatment followed by heat also inhibited protease activity in both the proteins (Fig. 4a, lanes 5 and 10). Thus all the three treatments individually inhibited protease activity of both trypsin and SAP.

Since we conjecture that the active site involved in phosphatase catalysis is also responsible for its protease action (Fig. 3a), any inhibitor of protease should result in the loss of phosphatase activity also. We tested the same with PMSF and trypsin inhibitor. Interestingly, these inhibitors resulted in the loss of phosphatase activity in all the three APs tested, where PMSF and trypsin inhibitor led to about 20% and 50% loss of AP activity, respectively (Fig. 4c). Further, we have demonstrated the increased mass (169.5 Da) of SAP by MALDI-mass spectrometry using AEBSF (4-(2Aminoethyl) benzenesulfonyl fluoride), a serine protease inhibitor (Fig. 4d). The expected increase in mass due to AEBSF binding is 183 Da. We chose AEBSF since it is more stable than PMSF, binds irreversibly to the active site serine and was also shown to inhibit 53% of the native phosphatase activity of SAP. Finally, we tested whether SAP can auto cleave itself. We found that the auto cleavage of SAP was specific to the condition where EDTA (10 mM), a metal ion chelator, was added (Fig. 4e). The auto cleavage activity of SAP in the absence of EDTA was much reduced. However, in the same conditions the protein showed a measurable extent of protease on a substrate protein added *in trans* (UVI31+) (Fig. 4a, lane 2).

We believe that SAP protease observed in the absence of EDTA is facilitated by an alternate serine (Ser89) that is unliganded by Zn^2+^ and therefore is free to perform proteolysis (Fig. 3b). The limited activity observed through this alternate serine perhaps reflects the sub-optimal substrate configuration of the protein tested. However, in the presence of EDTA both serines become catalytically available, thereby bringing about much more efficient auto cleavage. Nevertheless, we hypothesize that it is the alternate serine which is better placed than the liganded serine (Ser86) for the metal ion independent serine protease action.

Thus, as predicted by CLASP, we have uncovered an additional protease activity of SAP, acting not only on a trans substrate but also on itself and that its AP activity is inhibited by trypsin inhibitors.

## Discussion

### Catalytic Site Machinery

The specificity of active sites has been a long established fact. Over the last few decades studies have correlated their stringent spatial requirements with the electrostatic steps critical in the catalytic process [9], [16]–[19], [21], [24]. We elaborate this using two very well studied enzymatic activities - β-lactamases and serine proteases.

#### β-lactamases and Serine Proteases

**Figure 5:**
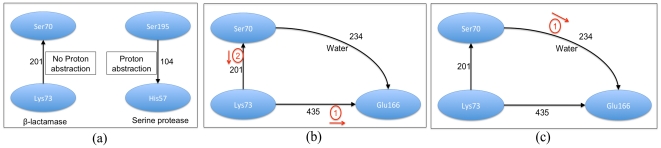
Proton abstraction in β-lactamases and serine proteases. The magnitude of the PD are annotated on the edges, the direction of the edge indicating the direction of the PD. (a) The differences in the way the nucleophilic serine is deprotonated in β-lactamases and serine proteases. Ser70 cannot donate proton to Lys73 because of reverse potential gradient in β-lactamase, but can do so in case of proteases (CLASP analysis, also see Discussion). (b) Mechanism in which the Lys73 abstracts a proton from Ser70 after it transfers a proton to Glu166. (c) Mechanism in which Glu166 abstracts a proton from Ser73 directly via a water molecule.

β-lactamases very efficiently catalyze the irreversible hydrolysis of the amide bond of the β-lactam ring, thus yielding biologically inactive products. The roles of Lys73 and Glu166 in the acylation step (Figure S3) as the catalytic base required to deprotonate the Ser70 is still controversial [48], [60]. While the important role of Lys73 in the catalysis is established by the high correlation in the PD between this residue and Ser70 in a set of β-lactamases in our current study (Table 1), the ambiguity on the role of Lys73 in deprotonating Ser70 as the sole base is evident from the reversed sign of the PD (Fig. 5a). The reversed sign here denotes that Lys73 by itself cannot act as the base required to abstract the proton from Ser70 (see discussion later).

Serine proteases cut peptide bonds in proteins using a well known catalytic triad [54]. The carboxylate side-chain of Asp hydrogen-bonds to a nitrogen-bonded proton on the His imidazole ring. This is possible because Asp is negatively charged at physiological pH. The other nitrogen on His hydrogen-bonds to the OH proton of Ser. This allows the nucleophilic oxygen atom of Ser to attack incoming substrates (peptide bonds). This step is similar to the deprotonation of Ser70 in β-lactamases. Though Ser, Asp and His are far apart in their primary sequence, they converge in the 3D structure to form the active site. The precise synchronized action between these residues is played out within a cleft in which the substrate fits in and is subsequently cleaved off.

#### Associating Electrostatics and the catalytic region

In 1923 Debye Huckel formulated a simple model for calculating the chemical potentials due to ionic species. The ions are considered smeared into a continuous distribution of charge, leading to a net charge density per unit volume. Water is considered as a continuous dielectric medium with a different dielectric constant than that of the protein. Current tools solve for the electrostatic potential using finite difference methods after assigning charge and radius parameters in a protein using a force field model.

Proteins possess ionizable groups and the *pKa* values of the ionizable residues in the active sites tend to be different from their intrinsic values [17], [21]. These large *pKa* deviations from standard values are used for the prediction of catalytic residues [19], [20]. *pKa* shifts are obtained by computing the difference in electrostatic energy of a charged residue with that of its neutral form -the resulting shift is added to the intrinsic *pKa* value [22], [61]. In contrast, our method relies on the PD between residues rather than the electrostatic energies in the active site. We have reported a high correlation in the electrostatic PD between pairs of residues. This feature has been exploited in CLASP to refine the matches obtained from spatial congruence to predict the catalytic residues.

We now discuss our results in the light of recent controversies associated with the acylation mechanisms proposed for β-lactamase activity. The reversal of PDs between two cognate pairs, namely Ser70-Lys73 and Ser195-His57 in β-lactamases and serine proteases respectively may be explained as follows. In both cases, the serine is rendered nucleophilic by deprotonation. This function is indisputably known to be carried out by the imidazole group (His) in serine proteases. However, the mechanism of proton abstraction and donation in β-lactamases by the active-site serine is still debated (Fig. 5a) [48], [60]. The PD between Ser195 and His57 in serine proteases is positive, thereby suggesting a high tendency of Ser to be deprotonated. This propensity is not apparent in β-lactamases, since the PD between Ser70 and Lys73 is negative implying that Lys73 could not possibly act as the sole general base, abstracting a proton from Ser70. However, it has been proposed that Lys73 could in fact abstract a proton only under the influence of Glu166 while forming the preacylation complex [62]. The high positive PD between Lys73 and Glu166 (Table S5) is consistent with the theory that Lys73 acts as the general base only after transferring a proton to Glu166 (Fig. 5b). Therefore, proton transfer from Ser70 to Lys73 is facilitated by the presence of Glu166 inspite of PD between Ser70 and Lys73 being negative. Induced fit imposed by the substrate binding is supposed to be instrumental in facilitating the directional proton abstraction initiated by Glu166 [63]. In the case of β-lactamases, such dramatic conformational changes in the course of this hydrolysis have been reported [64]. Another mechanism invokes Glu166 acting as the base responsible for proton abstraction via intervening water molecules [48], [65] (Fig. 5c). Our PD results are consistent with either mechanism, since the overall PD between Ser70 and Glu166 (independent of Lys73) is positive. The roles of Lys73 and Glu166 in the acylation step in β-lactamases have also been investigated using calculated *pKa* values [66], [67]. Lys73 would require an unusually low *pKa* value to act as a general base, while it has been experimentally shown that the *pKa* values of Lys73 is above 10 [68], thereby underscoring the importance of Glu166.

The statistical significance of the narrow range within which the electrostatic PD between pairs of residues in the active site falls needs to be established. One test of significance would be to find the potentials of all randomly occurring motifs of *SXXK*. The *SXX*K motif consists of the first two residues that we have used in defining the querying motif for β-lactamases (Ser70, Lys73, Ser130, Lys234). Clearly, these potentials should not trivially exhibit the patterns that we detect in β-lactamases. For the list of ∼3000 non-redundant proteins we extracted all the *SXXK* motifs and evaluated their PD (Figure S4). Numerically, approximately two-thirds of the values were found to lie outside the range for β-lactamases. When supplemented with distance congruence, these one-third proteins get pruned out further, thereby increasing the discriminatory power (Figure S2). It is to be noted that the querying motif for β-lactamases {Ser70, Lys73, Ser130, Lys234} offers a total of 6 residue pairs. Since CLASP integrates the PD scores of all 6 residue pairs, the final sensitivity would be significantly higher than what one obtains using only the {Ser70, Lys73} pair in the *SXX*K motif. This implies that more the number of residues in the motif, better is our accuracy in prediction.

### Probing a promiscuous protein using CLASP

The result of CLASP analysis on motifs extracted from CSA demonstrates its ability to classify any protein, putative or otherwise, with known structure (Figure S1 and Table 4). APs are one of the most widely researched promiscuous enzymes -they are known to have sulfate monoesterase, phosphate diesterase, and phosphonate monoesterase activities [69]–[71]. Recently another phosphite-dependent hydrogenase activity was also found in ECAP [72]. This hydrogenase activity is absent in APs from other organisms. Similarly other proteins from this superfamily show cross activity - for example the *Pseudomona*s *aeruginos*a arylsulfatase (PAS) which has the primary activity of hydrolyzing sulfate monoesters, also catalyzes the hydrolysis of phosphate monoesters [73]. On probing APs for additional promiscuous activities using all CSA motifs, we found that one of the APs, namely SAP (PDB id: 1K7H), showed good match with serine protease motif (Table 5a) among other matches. We tested the protease activity in SAP by *in vitro* experiments and corroborated the above prediction.

### Alkaline Phosphatases

APs are non-specific metalloenzymes that catalyze the hydrolysis of phosphomonoesters of various kinds into inorganic phosphate and alcohols [57]–[59]. They are found in several isoforms in prokaryotic, archeal and eukaryotic species, of which several crystal structures have been solved -SCAP (*Shewanella sp*), HSAP (*Halobacterium salinarum*), Tab5 AP (TAP; antarctic bacterium), Vibrio AP (VAP), human placenta AP (PLAP), ECAP (*Escherichia coli*) and shrimp AP (SAP) [57], [74]–[79]. While these enzymes have dissimilar sequences (Table S6) and structure, the central active-site comprising of three metal-binding sites is highly conserved.

The widely believed notion of “single enzyme, single substrate, single reaction” has been lately challenged by the observation that promiscuity is not that rare in enzymes [80], [81]. Promiscuity is now accepted as an advantageous feature for the divergent evolution of new catalysts [82]. While CLASP analysis detected various matching motifs in APs (Table 5a), we chose to test one of the better matches, namely serine protease. *In vitro* experiments showed that SAP indeed exhibits serine protease activity (Fig. 4a). However, the activity of SAP was vial dependent with some batches of SAP showing prominent activity while the others did not. To rule out any possibility of protease contamination in the commercial SAP, we purified it further by passing the protein through a 50 kDA centrifugal filter device followed by gel elution of a single polypeptide band corresponding to the size of SAP from an 8% Native PAGE gel by electroelution (Centrilutor microelectroelutor from Millipore). Gel elution is a technique used to extract a single polypeptide of the desired molecular weight from a polyacrylamide (SDS or Native) gel following electrophoresis. MALDI-TOF mass spectrometry of the purified SAP confirmed the presence of a single polypeptide of the expected size (55,263 Da) (Figure S5). We recovered protease activity even in the purified SAP (Figure S6), thereby ruling out any contribution by a contaminating extraneous protein. More convincingly the AP activity of all the APs tested (ECAP, SAP and CIAP) and across different batches was inhibited with trypsin inhibitor. This is conclusive evidence of the presence of a serine protease like scaffold in the active site of all the APs tested. Lack of protease activity in ECAP can be explained by the significant difference in PD between pairs of residues in ECAP from that of serine protease (Table 3)). It is a known fact that certain promiscuous activities are not universally present in all APs -for example, hydrogenase activity is present only in ECAP but not in other APs [72]. Since there are no known structures for CIAP, it is unclear why they do not exhibit protease activity. It has been shown recently that a cold AP responds differently to mutagenesis in the active sites as compared to ECAP due to intrinsic differences in the charge transfer network which is essential for the octahedral coordination in the catalytic site [83]. This might well explain why SAP, a cold enzyme, shows protease activity whereas the heat stable APs (ECAP and CIAP) do not.

The phosphatase activity of AP is known to decline by protease treatment [84]. We speculate that auto protease activity may have evolved in APs to regulate the level of phosphatase activity, which is known to change dramatically under various physiological conditions. Auto cleavage of AP in the absence of metal ions (Fig. 4e) is a good switch from AP to protease, since APs are rendered non-functional in the absence of metal ions.

The AP superfamily includes members like nucleotide pyro-phosphatases/phosphodiesterases (NPPs), cofactor-independent phosphoglycerate mutases (iPGMs), phosphonoacetate hydrolases, phosphonate monoester hydrolases (PMHs) and arylsulfatases (ASs) apart from APs, and is inferred based on structural homology [85]–[87]. However, the active site residues in the superfamily are quite distinct - AS and PMH have one metal ion, NPP and iPGM have two while the APs have 3 metal ions [88], [89]. It will be insightful to apply CLASP analysis on these divergent enzymes for a protease motif. The result of such an analysis reveals that NPPs and APs are likely candidates to show protease activity (Table 5b), and that they are more strongly related to each other than other proteins in the superfamily.

### Limitations, Future Directions and Conclusions

An inherent limitation of CLASP is its inability to distinguish between mirror images. This is typical of methods that use RMSD. Another shortcoming in CLASP is that it is limited to single polypeptide chains in proteins. Composite active sites using different subunits cannot be handled currently. This limitation can be overcome with degraded runtimes. Complex active sites comprising of several residues can in principle be analyzed provided one specifies the complete set of involved residues. Once again, this involves a runtime penalty. One must note that CLASP relies only on static parameters associated with protein structures and does not include any dynamics associated with catalysis. Integrating methods such as molecular dynamics simulations based on quantum mechanical calculations with CLASP based predictions will improve the accuracy of detecting catalytic sites that rely heavily on substrate induced dynamics [90], [91].

CLASP raises a few important points that need to be investigated further. The relationship between activity rates and the physical 3D signature and electrostatic properties is something that needs to be probed in depth. CLASP provides us with an ordered set of proteins, which exhibit a particular enzymatic function. An intriguing question is whether this ordering would also apply to the *kca*t values of these proteins. Previous work in our laboratory has established that the protein UVI31+ has weak β-lactamase activity, although sequence alignment with known β-lactamases has failed to identify the active site [92]. CLASP based predictions and site directed mutagenesis are underway to ascertain the active site.

Engineering enzymes to mimic evolution has been previously applied to obtain promising results [93], [94]. A pioneering work has shown that four amino acid substitutions convert an oleate 12-desaturase to a hydroxylase [95]. Similarly, trypsin has been converted to work as chymotrypsin by suitably altering the surface loops [96]. Such work typically chooses enzymes with weak promiscuous activity and go on to strengthen this activity through site directed mutations. CLASP provides the perfect platform to check the viability of such an effort in any protein for any given activity. A more challenging task is to alter an enzyme in such a way that a new activity is imparted. CLASP can achieve this with improvised algorithms. We are implementing such a task on *E. coli* bolA protein [97] that shares strong domain homology with UVI31+, but lacks β-lactamase activity. A web server is also being designed to allow users the ability to run CLASP on their protein of interest.

CLASP integrates the features that are most critical to the active site machinery - spatial configuration and electrostatic properties - to identify active sites in proteins. Spatial congruence reveals putative matches. These are refined based on the observation that a strong correlation exists in the electrostatic potentials among residues in active sites for any particular activity. This is corroborated by data on the PD in pairs of residues for a set of proteins from the same family showing very small standard deviation. In addition, CLASP also takes into account the diversity in protein configurations that account for the same enzymatic action. CLASP can be run quickly on known motifs to classify proteins with unknown functions. Promiscuous functions unearthed using CLASP should be substantiated by *in vitro* tests in tandem. We have unravelled a promiscuous proteolytic activity in SAP using our method, which has been validated here using *in vitro* experiments.

## Methods

Table S7 lists the datasets of the proteins used.

### Flow

CLASP starts with a motif of the active sites. First all sets of n residues with the constraint that each residue should be from the group specified are obtained (Figure S7). To obtain matches for the reference motif in the query protein, we use an exhaustive search procedure for the pattern similar to the one used in SPASM [25] taking advantage of the labelling data with the motif and the protein to prune the search space. The search space is limited to a user defined distance, such that all residues in a given match are within this distance from each other. Another similar technique is also frequently used but shares the same limitations with motif sizes [98]. Other techniques are able to handle longer motif searches [99]. Since CLASP has quick runtimes, revising the algorithm was not required. For each such match, the 3D distances and PD are computed. These values are then compared with the corresponding feature values obtained from the reference protein and adjusted with the user defined weights to obtain a score. The distance scores are normalized to take in account the fact that the same pair wise distance deviation should count more when the reference distance is less. For example, a deviation of 1 Å is more significant if the distance between 2 residues is 4 Å, than if the distance between them is 8 Å in the reference protein. In order to score potential differences, we ignore deviations if the potential difference in the cognate pair in the query and the reference residue pair are both close to 0. Similarly for higher potential differences, the deviations are more loosely constrained than for lower potential differences. The differences are absolute values. The best scoring match is considered as the representative for that protein and defines our predicted catalytic site which can be subjected to mutational analysis for verification. Thus each motif represents a function to be searched within a protein of unknown function. A motif can be manually provided (Fig. 1). By automatically extracting all specified motifs from the Catalytic Site Atlas (CSA) database (Figure S1), we can query an unknown protein for all listed functions (www.sanchak.com/CLASP).

### Calculating electrostatic potential difference

Adaptive Poisson-Boltzmann Solver (APBS) package was used to calculate the PD between the reactive atoms of the corresponding proteins [14] using the PARSE force field [100]. Assigning charge and radius parameters in a protein using a force field model, current tools solve for the electrostatic potential using finite difference methods [15]. PDB2PQR calculates the grid dimensions, grid spacing, grid lengths and grid centre, and the charges on the atoms [101] using the PARSE force field [100]. The charge assignment is static -for example the OG atom of Ser always has a charge of −0.49e. However, the potential on this atom will depend on how it is placed with respect to other atoms in the protein. The PBE is solved using a lattice representation where the charges and dielectric properties are mapped on the cubic lattice in the so called grid points where the PD is calculated. The APBS parameters are set as follows -solute dielectric: 2.000, solvent dielectric: 78, solvent probe radius: 1.4 Å, Temperature: 298 K and 0 ionic strength. A method that has created a database of pre-calculated values reported on a per residue basis for all PDB protein structures suggests a grid spacing of 0.4 Å [102]. We have used the PDB2PQR calculated grid spacing which varies from 0.4 Å to 0.6 Å. The electrostatic potential is in dimensionless units of kT/e where k is Boltzmann's constant, T is the temperature in K and e is the charge of an electron. The grid potential value depends quite strongly on the grid spacing and on the system position relative to the lattice [102]. Other sources of variations are discretization of the continuously-specified problem coefficients. However, the potential differences and most other observables (like solvation energies or forces) rely on the difference in two calculations on the same grid, This eliminates most of the grid-sensitivity for sufficiently fine grids. The largest deviations in potential differences are found inside clefts and indentations that occur on the dielectric boundaries, as in the active sites [103]. Nonetheless the correlation between the potential differences measured in dimensionless units is apparent and this correlation of PD is used in our method for screening the active sites.

### Protein, Substrate and Reagents

SAP was purchased from USB, CIAP from Gibco BRL and ECAP from Sigma. PMSF (Phenylmethylsulfonyl fluoride) and trypsin inhibitor from chicken egg white was obtained from Roche. pNPP (p-Nitrophenyl phosphate Disodium salt Hexahydrate) was obtained from SRL. We used trypsin from Sigma and the 50 kDa centrifugal filter device from Millipore. AEBSF used was from Calbiochem.

### Protease Assay

Each reaction mixture (30 µl total volume) contained 13 µM of purified UVI31+ protein [92] (14 kDa) and 3 units of AP in 50 mM ammonium bicarbonate, followed by overnight incubation at 37°C. The protein was then denatured by the addition of 7 µl of SDS-denaturing solution (200 mM Tris-HCl pH 6.8, 8% SDS, 40% Glycerol, 4% 2-mercaptoethanol, 50 mM EDTA pH 8.0, and 0.08% bromophenol blue) and heating it at 100°C for 3 min. The sample was subjected to 15% SDS-PAGE analysis followed by staining with Coomassie Brilliant Blue and band quantification using Image J. For the inhibition of protease activity of SAP, three different conditions were employed i.e., (i) 0.1% SDS followed by heating at 100°C for 5 min, (ii) 1 mM PMSF, and (iii) 500 ng/ml of trypsin inhibitor, before substrate addition. UVI31+ protein (13 µM) was then added as the substrate and residual enzyme activity was measured.

### Alkaline Phosphatase Assay

Reaction of AP (1 unit) with p-nitrophenylphosphate (0.2 mM) as substrate, in a total volume of 200 µl was set up in 50 mM ammonium bicarbonate buffer, and incubated at room temperature for 2 min. Absorbance at 405 nm was measured against deionized water. Inhibition studies were performed as mentioned above for protease assay.

### MALDI-TOF Analysis

MALDI-TOF mass spectrometric analysis was performed using an UltraFlex II TOF/TOF of Bruker Dal-tonics (Germany). Positive ionization and linear mode were used. The experimental parameters are as follows: laser power 60%, voltage 25 kV, and the mass difference in linear mode with external calibration of the machine (Ultraflex II, Bruker Daltonics) is less than ±100 ppm i.e. less than ±0.01%. The matrix was sinapinic acid. The external calibration standard consisted of insulin, ubiquitin, cytochrome C and myoglobin.

### External Tools

We extensively integrated and used the freely available BioPerl [104] modules and Emboss [105] tools. All protein structures were rendered by PyMol (http://www.pymol.org/).

### CSA Motif extraction

The Catalytic Site Atlas (CSA), available online, provides catalytic residue annotation for enzymes in the PDB [35]. The database consists of two types of annotated sites: an original hand-annotated set containing information extracted from the primary literature and a homologous set containing residues inferred by PSIBLAST [56]. We downloaded the file *CS*A_2_2_12.*lis*t from the CSA site and extracted about ∼400 motifs. The motifs picked were those that were extracted from literature, had 3, 4 or 5 residues and were all confined to one polypeptide. Multiple chain active sites (those shared by subunits) were ignored.

### Runtimes

The runtimes for CLASP are presented in Figure S8. The typical run time for a single protein is about an hour. A cluster of 128 processors has been used to implement parallel processing on mutually exclusive aspects of the program.

## Supporting Information

Figure S1 The flow for assigning functions to putative proteins based on the motifs in the CSA Database. Any unknown protein is compared with each motif. Precomputed values help to achieve quick runtimes. Parallel computation has been possible through a cluster of 128 processors to speed up this process due to the mutually exclusive nature of each comparison.
(PDF)

Figure S2 ∼500 non-redundant proteins that have the best 3D matches with the Class A β-lactamase motif. We demonstrate that electrostatic conformity leads to a considerable reduction in false positives. The dashed line denotes the highest scoring β -lactamase (PDB id: 2QZ6). When 3D conformity is the sole criteria, there are 80% of the proteins that are below this score, and qualify as possible β -lactamases. This number reduces to 20% when electrostatic conformity is combined with 3D congruence. This analysis was performed on a set of ∼500 proteins that were the best candidates using a quick 3D match on all the ∼50,000 proteins in the PDB database. It is to be noted that the 4 fold enrichment achieved on this set is an underestimation of the power of our method since the chosen set of proteins was a filtered category through 3D congruence.
(PDF)

Figure S3 The overall pathway for β -lactam hydrolysis. There are two main steps - acylation and hydrolysis. The acylation is common to β-lactamases and penicillin-binding proteins (PBP). Thus antibiotic resistance essentially arises from the deacylation reaction. Starting with the ground state (a) and passing through a high energy acylation state (b) the acyl-enyzme intermediate is formed (c) due to the nucleophilic attack of the serine on the β-lactam. The next step is hydrolysis (d) and finally the product is formed (e), and the enzyme is ready for another cycle.
(PDF)

Figure S4 The potential difference between the Ser and Lys residues in all the SXXK motifs (in a sample of 1500 motifs where each one is numbered arbitrarily) found in the ∼3000 non-redundant proteins.
(PDF)

Figure S5 MALDI mass spectra of the purified SAP. The small peak at approximately 28 kDa represents the doubly charged molecular ion at half the m/z value.
(PDF)

Figure S6 Protease activity of SAP after purification. Substrate protein (UVI31+; lane 1) was incubated overnight at 37°C with stock SAP (lane 2) and purified SAP (lane 3). Purification was done by passing the protein through a 50 kDA centrifugal filter device followed by gel elution of a single polypeptide band corresponding to the size of SAP from a 8% native PAGE gel by electroelution (Centrilutor micro-electroelutor from Millipore).
(PDF)

Figure S7 Algorithm ScoreSingleProtein - score any given protein for enzymatic function *F*.
(TIF)

Figure S8 (a): Runtimes for running CLASP. The runtimes have been divided into the three most time intensive parts. - the 3D matching, running PDB2PQR which assigns charges on the proteins and APBS which calculates calculates the potential. (b): Runtimes when each of ∼50 putative proteins was run on ∼400 motifs that were automatically extracted from the CSA Database.
(PDF)

Table S1 Config File: Input to CLASP which allows the user to set the reactive atoms for each amino acid, and to control features of stereochemically equivalent amino acid side chains in the active sites and thus obtain acceptable values of sensitivity and specificity. Motif in β-lactamases – and grouping: We have taken the motif {Ser70, Lys73, Ser130, Lys234} from a Class A β-lactamase (PDB id: 1G68) to represent β-lactamases. For example the group Lysgrp has three residues {Lys, Arg, His}, whereas the more restricted group Lysonly has only one residue Lys.
(PDF)

Table S2 (a) APBS calculated potential differences in β-lactamases when queried using a Class A β-lactamase motif. (b) APBS calculated potential differences in residue pairs in penicillin-binding proteins (PBP). Potential differences are in dimensionless units of kT/e (k is Boltzmann's constant, T is the temperature in K and e is the charge of an electron).
(PDF)

Table S3 Predicted residues and pairwise distances for a list of Class A, C and D β-lactamases using a Class A β-lactamase motif {Ser70, Lys73, Ser130, Lys234}. The distances are specified in the reference protein (PDB id: 2G2U) in A. For the remaining, we show the deviation from the reference value. In the Class C β-lactamases (2QZ6 for example), Ser is replaced by the stereochemically equivalent Tyr in the third position of the match. However, in some of the Class C β-lactamases (2QZ6 for example), the predicted Tyr (Tyr112) is different from the one known to be responsible for catalysis (Tyr150). The inherent shortcoming of any method that uses RSMD is its inability to distinguish between two mirror image configurations. This is evident for the Class A β-lactamase 3DW0 and the Class D β-lactamase 2HP5, which is a mirror image configuration with respect to other motifs in this set. It can also be seen that the Class A proteins match better than the Class C and Class D proteins, since we are using a Class A β-lactamase motif. One Class C β-lactamase (PDB id: 3GQZ) is an exception, possibly because this protein has had conformational changes due to fragment binding.
(PDF)

Table S4 Pairwise distances and potential differences between pairs of residues in the reference motif from the demaminase (PDB id: 1CD5) (Residues listed in CSA database: {His143,Glu148,Asp141,Asp72}) and the unknown protein Tm0936 from *Thermotoga maritima* (PDB id: 1P1M) (CLASP predicted residues: {His55,Glu203,Asp279,Asp113}).
(PDF)

Table S5 Identity/Similarity among all Aps.
(PDF)

Table S6 Potential difference between Lys73 and Glu166 for a motif from a Class A β-lactamase which now includes the Glu166 for a list of Class A β-lactamase proteins {Ser70, Lys73, Ser130, Lys234, Glu166}, the high potential differences observed are consistent with the theory that Lys73 is protonated in the initial stages, and acts as the general base to Ser70 only after transferring a proton to the Glu166.
(PDF)

Table S7 Dataset.
(PDF)
