# Supplementary material for: Active Site Detection by Spatial Conformity and Electrostatic Analysis—Unravelling a Proteolytic Function in Shrimp Alkaline Phosphatase
Source: PLoS One. 2011 Dec 8;6(12):e28470. doi: 10.1371/journal.pone.0028470 (PMC3234256; doi:10.1371/journal.pone.0028470)
Supplement: Figure S1 — The flow for assigning functions to putative proteins based on the motifs in the CSA Database. Any unknown protein is compared with each motif. Precomputed values help to achieve quick runtimes. Parallel computation has been possible through a cluster of 128 processors to speed up this process due to the mutually exclusive nature of each comparison. (PDF) [file pone.0028470.s001.pdf]

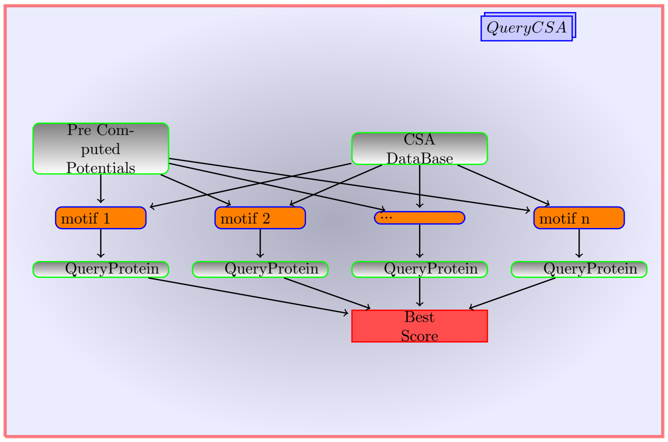

Supplementary Fig. 1: The flow for assigning functions to putative proteins based on the motifs in the CSA Database. Any unknown protein is compared with each motif. Precomputed values help to achieve quick runtimes. Parallel computation has been possible through a cluster of 128 processors to speed up this process due to the mutually exclusive nature of each comparison.
