# Supplementary material for: Active Site Detection by Spatial Conformity and Electrostatic Analysis—Unravelling a Proteolytic Function in Shrimp Alkaline Phosphatase
Source: PLoS One. 2011 Dec 8;6(12):e28470. doi: 10.1371/journal.pone.0028470 (PMC3234256; doi:10.1371/journal.pone.0028470)
Supplement: Figure S2 — ∼500 non-redundant proteins that have the best 3D matches with the Class A β-lactamase motif. We demonstrate that electrostatic conformity leads to a considerable reduction in false positives. The dashed line denotes the highest scoring β -lactamase (PDB id: 2QZ6). When 3D conformity is the sole criteria, there are 80% of the proteins that are below this score, and qualify as possible β -lactamases. This number reduces to 20% when electrostatic conformity is combined with 3D congruence. This analysis was performed on a set of ∼500 proteins that were the best candidates using a quick 3D match on all the ∼50,000 proteins in the PDB database. It is to be noted that the 4 fold enrichment achieved on this set is an underestimation of the power of our method since the chosen set of proteins was a filtered category through 3D congruence. (PDF) [file pone.0028470.s002.pdf]

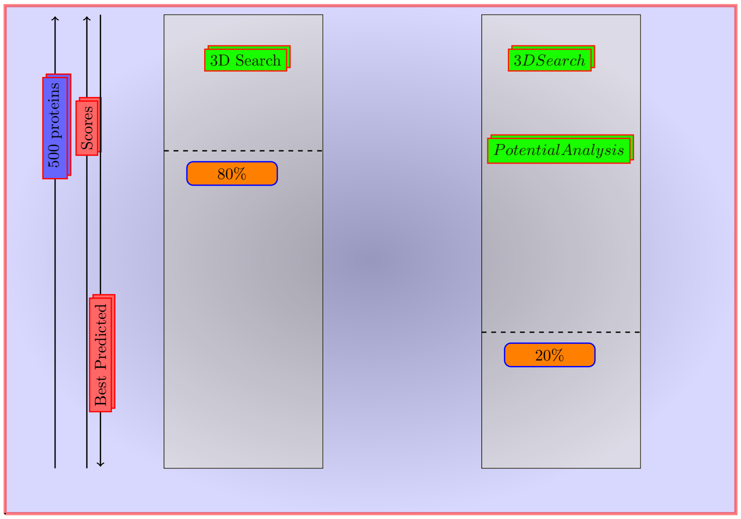

Supplementary Fig. 2: ∼500 non-redundant proteins that have the best 3D matches with the Class A β-lactamase motif. We demonstrate that electrostatic conformity leads to a considerable reduction in false positives. The dashed line denotes the highest scoring β-lactamase (PDB id: 2QZ6). When 3D conformity is the sole criteria, there are 80% of the proteins that are below this score, and qualify as possible β-lactamases. This number reduces to 20% when electrostatic conformity is combined with 3D congruence. This analysis was performed on a set of ∼500 proteins that were the best candidates using a quick 3D match on all the ∼50,000 proteins in the PDB database. It is to be noted that the 4 fold enrichment achieved on this set is an underestimation of the power of our method since the chosen set of proteins was a filtered category through 3D congruence.
