# Supplementary material for: Active Site Detection by Spatial Conformity and Electrostatic Analysis—Unravelling a Proteolytic Function in Shrimp Alkaline Phosphatase
Source: PLoS One. 2011 Dec 8;6(12):e28470. doi: 10.1371/journal.pone.0028470 (PMC3234256; doi:10.1371/journal.pone.0028470)
Supplement: Figure S3 — The overall pathway for β -lactam hydrolysis. There are two main steps - acylation and hydrolysis. The acylation is common to β-lactamases and penicillin-binding proteins (PBP). Thus antibiotic resistance essentially arises from the deacylation reaction. Starting with the ground state (a) and passing through a high energy acylation state (b) the acyl-enyzme intermediate is formed (c) due to the nucleophilic attack of the serine on the β-lactam. The next step is hydrolysis (d) and finally the product is formed (e), and the enzyme is ready for another cycle. (PDF) [file pone.0028470.s003.pdf]

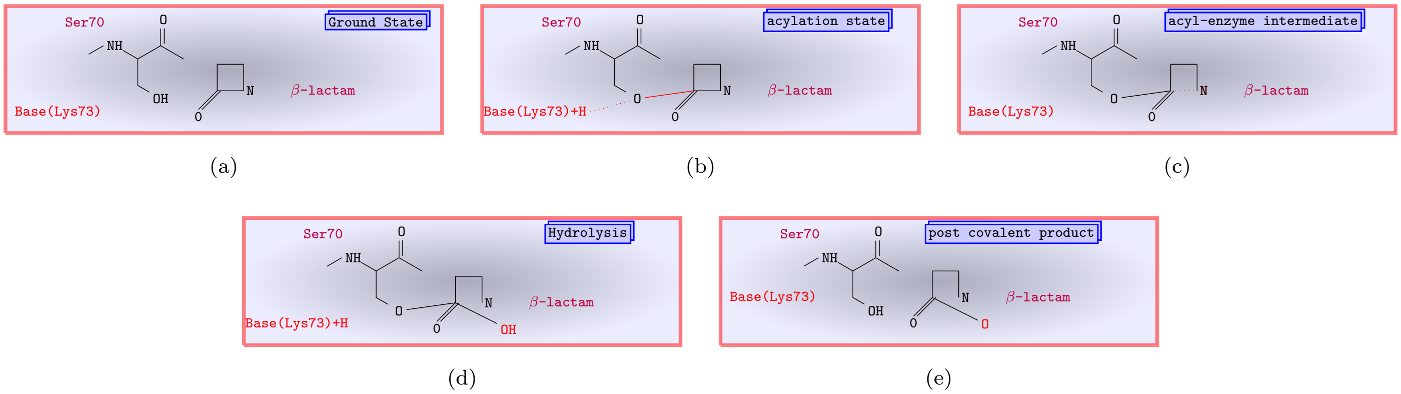

Supplementary Fig. 3: **The overall pathway for β-lactam hydrolysis** There are two main steps - acylation and hydrolysis. The acylation is common to β-lactamases and penicillin-binding proteins (PBP). Thus antibiotic resistance essentially arises from the deacylation reaction. Starting with the ground state (**a**) and passing through a high energy acylation state (**b**) the acyl-enyzme intermediate is formed (**c**) due to the nucleophilic attack of the serine on the β-lactam. The next step is hydrolysis (**d**) and finally the product is formed (**e**), and the enzyme is ready for another cycle.
