# Supplementary material for: Active Site Detection by Spatial Conformity and Electrostatic Analysis—Unravelling a Proteolytic Function in Shrimp Alkaline Phosphatase
Source: PLoS One. 2011 Dec 8;6(12):e28470. doi: 10.1371/journal.pone.0028470 (PMC3234256; doi:10.1371/journal.pone.0028470)
Supplement: Figure S4 — The potential difference between the Ser and Lys residues in all the SXXK motifs (in a sample of 1500 motifs where each one is numbered arbitrarily) found in the ∼3000 non-redundant proteins. (PDF) [file pone.0028470.s004.pdf]

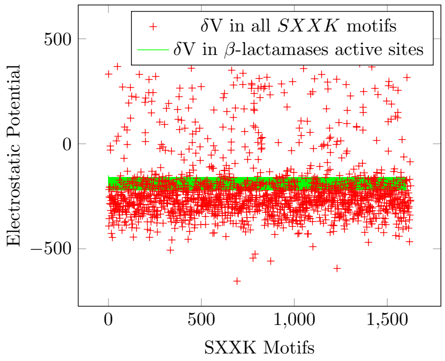

Supplementary Fig. 4: The potential difference between the Ser and Lys residues in all the $SXXK$ motifs (in a sample of 1500 motifs where each one is numbered arbitarily) found in the ∼3000 non-redudant proteins.
