# Supplementary material for: Active Site Detection by Spatial Conformity and Electrostatic Analysis—Unravelling a Proteolytic Function in Shrimp Alkaline Phosphatase
Source: PLoS One. 2011 Dec 8;6(12):e28470. doi: 10.1371/journal.pone.0028470 (PMC3234256; doi:10.1371/journal.pone.0028470)
Supplement: Figure S5 — MALDI mass spectra of the purified SAP. The small peak at approximately 28 kDa represents the doubly charged molecular ion at half the m/z value. (PDF) [file pone.0028470.s005.pdf]

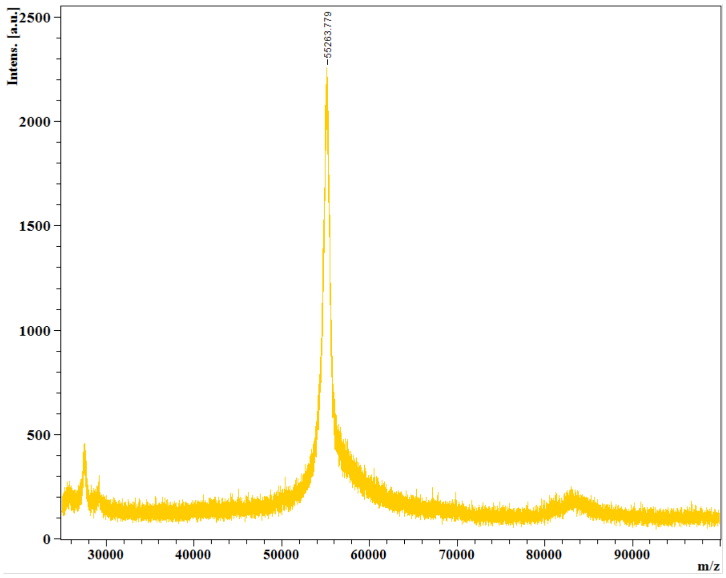

Supplementary Fig. 5: MALDI mass spectra of the purified SAP. The small peak at approximately 28 kDa represents the doubly charged molecular ion at half the m/z value.
