# Supplementary material for: Active Site Detection by Spatial Conformity and Electrostatic Analysis—Unravelling a Proteolytic Function in Shrimp Alkaline Phosphatase
Source: PLoS One. 2011 Dec 8;6(12):e28470. doi: 10.1371/journal.pone.0028470 (PMC3234256; doi:10.1371/journal.pone.0028470)
Supplement: Figure S6 — Protease activity of SAP after purification. Substrate protein (UVI31+; lane 1) was incubated overnight at 37°C with stock SAP (lane 2) and purified SAP (lane 3). Purification was done by passing the protein through a 50 kDA centrifugal filter device followed by gel elution of a single polypeptide band corresponding to the size of SAP from a 8% native PAGE gel by electroelution (Centrilutor micro-electroelutor from Millipore). (PDF) [file pone.0028470.s006.pdf]

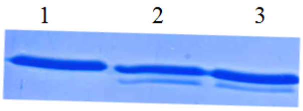

Supplementary Fig. 6: Protease activity of SAP after purification. Substrate protein (UVI31+; lane 1) was incubated overnight at 37 °C with stock SAP (lane 2) and purified SAP (lane 3). Purification was done by passing the protein through a 50 kDA centrifugal filter device followed by gel elution of a single polypeptide band corresponding to the size of SAP from a 8% native PAGE gel by electroelution (Centrilutor micro-electroelutor from Millipore).
