# Supplementary figures and images for: Active Site Detection by Spatial Conformity and Electrostatic Analysis—Unravelling a Proteolytic Function in Shrimp Alkaline Phosphatase
Source: PLoS One. 2011 Dec 8;6(12):e28470. doi: 10.1371/journal.pone.0028470 (PMC3234256; doi:10.1371/journal.pone.0028470)

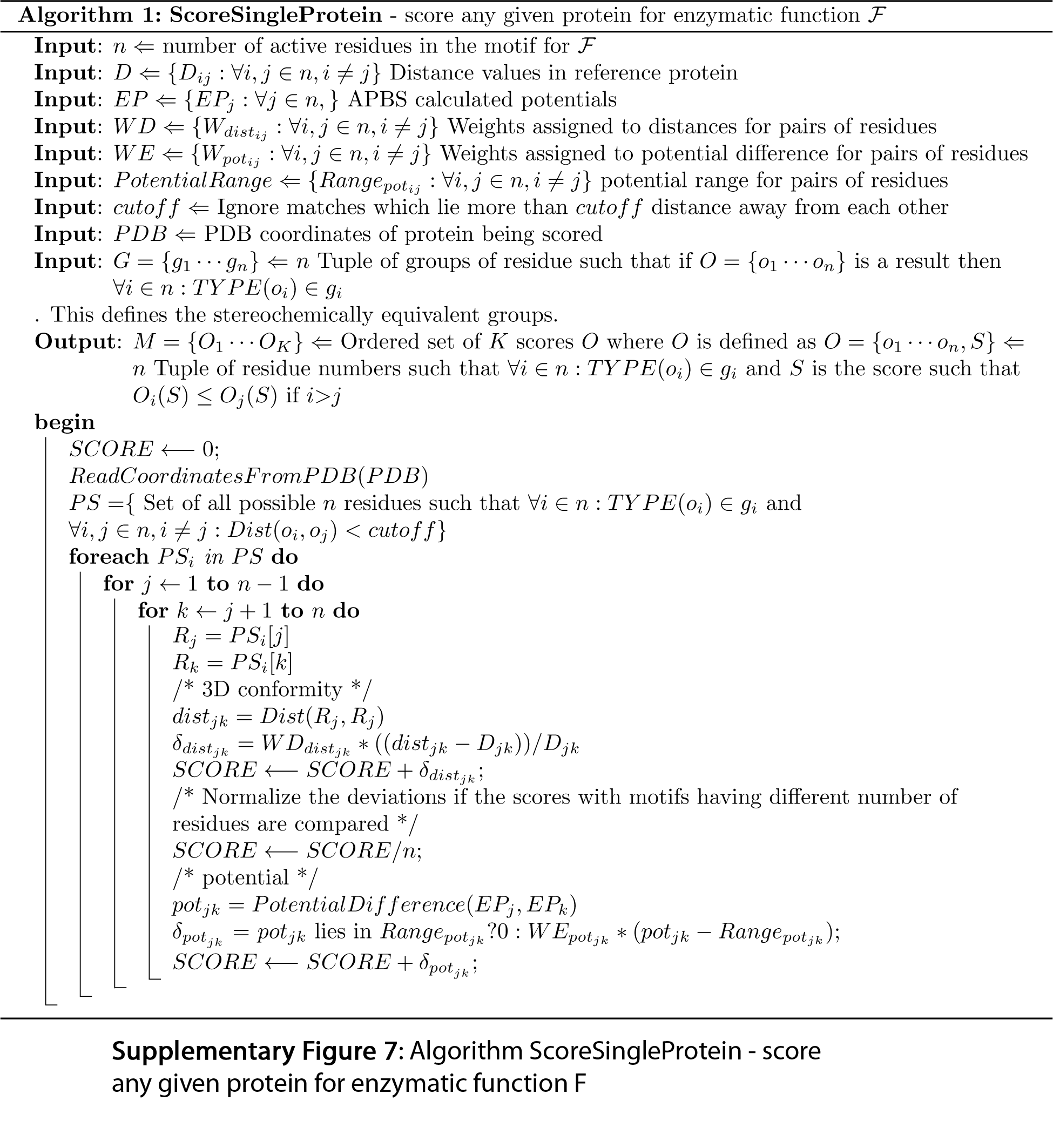

Supplement: Figure S7 — Algorithm ScoreSingleProtein - score any given protein for enzymatic function F. (TIF) [file pone.0028470.s007.tif]
