# Supplementary material for: Active Site Detection by Spatial Conformity and Electrostatic Analysis—Unravelling a Proteolytic Function in Shrimp Alkaline Phosphatase
Source: PLoS One. 2011 Dec 8;6(12):e28470. doi: 10.1371/journal.pone.0028470 (PMC3234256; doi:10.1371/journal.pone.0028470)
Supplement: Figure S8 — (a): Runtimes for running CLASP. The runtimes have been divided into the three most time intensive parts. - the 3D matching, running PDB2PQR which assigns charges on the proteins and APBS which calculates calculates the potential. (b): Runtimes when each of ∼50 putative proteins was run on ∼400 motifs that were automatically extracted from the CSA Database. (PDF) [file pone.0028470.s008.pdf]

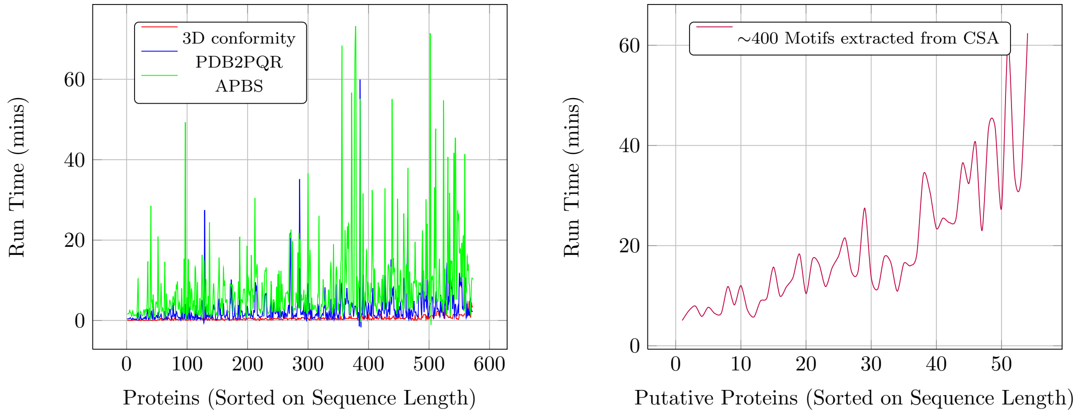

(a) (b)

Supplementary Fig. 8: **(a)**: Runtimes for running CLASP. The runtimes have been divided into the three most time intensive parts - the 3D matching, running PDB2PQR which assigns charges on the proteins and APBS which calculates the potential. **(b)**: Runtimes when each of ∼50 putative proteins was run on ∼400 motifs that were automatically extracted from the CSA Database.
