# Supplementary material for: Active Site Detection by Spatial Conformity and Electrostatic Analysis—Unravelling a Proteolytic Function in Shrimp Alkaline Phosphatase
Source: PLoS One. 2011 Dec 8;6(12):e28470. doi: 10.1371/journal.pone.0028470 (PMC3234256; doi:10.1371/journal.pone.0028470)
Supplement: Table S1 — Config File: Input to CLASP which allows the user to set the reactive atoms for each amino acid, and to control features of stereochemically equivalent amino acid side chains in the active sites and thus obtain acceptable values of sensitivity and specificity. Motif in β-lactamases – and grouping: We have taken the motif {Ser70, Lys73, Ser130, Lys234} from a Class A β-lactamase (PDB id: 1G68) to represent β-lactamases. For example the group Lysgrp has three residues {Lys, Arg, His}, whereas the more restricted group Lysonly has only one residue Lys. (PDF) [file pone.0028470.s009.pdf]

Supplementary Table. 1: **Config File**: Input to CLASP which allows the user to set the reactive atoms for each amino acid, and to control features of stereochemically equivalent amino acid side chains in the active sites and thus obtain acceptable values of sensitivity and specificity. **Motif in β-lactamases - and grouping**: We have taken the motif {Ser70, Lys73, Ser130, Lys234} from a Class A β-lactamase (PDB id: 1G68) to represent β-lactamases. For example the group Lys_{grp} has three residues {Lys, Arg, His}, whereas the more restricted group Lys_{only} has only one residue Lys.

reactive atoms	ATOMCONFIG	LYS	NZ				
	ATOMCONFIG	SER	OG				
	ATOMCONFIG	THR	OG1				
	ATOMCONFIG	CYS	SG				
	ATOMCONFIG	ASN	ND2				
	ATOMCONFIG	GLN	NE2				
	ATOMCONFIG	ARG	NH1				
	ATOMCONFIG	HIS	ND1				
	ATOMCONFIG	TYR	OH				
	ATOMCONFIG	TRP	NE1				
	ATOMCONFIG	ASP	OD1				
	ATOMCONFIG	GLU	OE1				
	ATOMCONFIG	TYR	OH				
	ATOMCONFIG	VAL	CG1				
	ATOMCONFIG	GLY	CA				
	ATOMCONFIG	ALA	CB				
	ATOMCONFIG	LEU	CD1				
	ATOMCONFIG	MET	SD				
	ATOMCONFIG	MET	SD				
	ATOMCONFIG	ILE	CD1				
	ATOMCONFIG	PHE	CA				
	ATOMCONFIG	HIP	CA				
	ATOMCONFIG	PRO	CA				
grouping	GROUP	ALAONLY	ALA				
	GROUP	ARGONLY	ARG				
	GROUP	ASNONLY	ASN				
	GROUP	ASPONLY	ASP				
	GROUP	LEUONLY	LEU				
	GROUP	LYSONLY	LYS				
	GROUP	METONLY	MET				
	GROUP	PHEONLY	PHE				
	GROUP	PROONLY	PRO				
	GROUP	SERONLY	SER				
	GROUP	THRONLY	THR				
	GROUP	TRPONLY	TRP				
	GROUP	TYRONLY	TYR				
	GROUP	VALONLY	VAL				
	GROUP	SERGRP	SER	THR	CYS	ASN	GLN
	GROUP	LYSGRP	LYS	ARG	HIS		
	GROUP	GLYGRP	VAL	LEU	GLY	ALA	MET
	GROUP	TYRGRP	TYR TRP				
	GROUP	GLUGRP	GLU ASP				
	GROUP	GLUTYR	GLU TYR				
	Motif	SER70	LYS73	SER130	LYS234		
Controlling sensitivity	Least constrained	Ser_{only}	Lys_{grp}	Ser_{grp}	Lys_{grp}		
	More constrained	Ser_{only}	Lys_{only}	Ser_{grp}	Lys_{grp}		
	Maximum constrained	Ser_{only}	Lys_{only}	Ser_{only}	Lys_{only}		
