# Supplementary material for: Active Site Detection by Spatial Conformity and Electrostatic Analysis—Unravelling a Proteolytic Function in Shrimp Alkaline Phosphatase
Source: PLoS One. 2011 Dec 8;6(12):e28470. doi: 10.1371/journal.pone.0028470 (PMC3234256; doi:10.1371/journal.pone.0028470)
Supplement: Table S2 — (a) APBS calculated potential differences in β-lactamases when queried using a Class A β-lactamase motif. (b) APBS calculated potential differences in residue pairs in penicillin-binding proteins (PBP). Potential differences are in dimensionless units of kT/e (k is Boltzmann's constant, T is the temperature in K and e is the charge of an electron). (PDF) [file pone.0028470.s010.pdf]

Supplementary Table. 2: **(a)** APBS calculated potential differences in β-lactamases when queried using a Class A β-lactamase motif. **(b)** APBS calculated potential differences in residue pairs in penicillin-binding proteins (PBP). Potential differences are in dimensionless units of kT/e (k is Boltzmann's constant, T is the temperature in K and e is the charge of an electron).

	PDB id	S70/K73 OG/NZ	S70/S130 OG/OG	S70/R234 OG/NH1	K73/S130 NZ/OG	K73/R234 NZ/NH1	S130/R234 OG/NH1
a	2G2U(A)	-201.7	22.3	-250.3	224.0	-48.6	-272.7
	2QZ6(C)	-196.5	15.9	-240.3	212.4	-43.7	-256.1
	2HP5(D)	-215.1	-12.8	-228.3	202.3	-13.2	-215.5
	1BUL(A)	-237.3	21.0	-273.1	258.3	-35.8	-294.0
	1DY6(A)	-192.2	15.5	-245.4	207.8	-53.2	-261.0
	1GA0(C)	-273.8	-28.2	-291.7	245.6	-17.9	-263.5
	1O7E(A)	-195.2	14.5	-204.6	209.6	-9.4	-219.1
	2WZZ(C)	-248.3	38.8	-293.1	287.1	-44.8	-331.9
	2ZC7(C)	-195.5	-11.5	-212.1	184.0	-16.6	-200.6
	3GQZ(C)	-276.1	-16.2	-241.8	259.9	34.3	-225.6
	4BLM(A)	-192.9	-1.3	-209.5	191.6	-16.6	-208.2
	1JTG(A)	-192.7	36.7	-203.9	229.4	-11.2	-240.7
	3DW0(A)	-164.8	21.5	-190.7	186.4	-25.9	-212.2
	1FOF(D)	-246.4	90.1	-246.0	336.5	0.4	-336.2
	Mean	-219.4	4.2	-242.2	223.6	-22.8	-246.4
	SD	28.9	19.0	29.7	29.6	24.9	36.8
b	3HUM(PBP4)	-184.0	35.2	-202.2	219.1	-18.2	-237.3
	1QME(PBP2X)	-211.5	-38.2	-242.0	173.3	-30.5	-203.8
	3A3F(PBP4)	-192.8	21.7	-183.8	214.5	9.0	-205.5
	1W5D(PBP4)	-216.3	-22.5	-269.3	193.8	-53.1	-246.8
	3FWM(PBP1B)	-211.3	32.5	-105.4	243.8	105.8	-137.9
	1SKF(PBP)	-207.9	27.3	-199.7	235.2	8.2	-226.9
	Mean	-203.2	5.7	-200.5	208.9	2.6	-206.3
	SD	12.5	30.2	56.2	23.9	55.4	38.2
