# Supplementary material for: Active Site Detection by Spatial Conformity and Electrostatic Analysis—Unravelling a Proteolytic Function in Shrimp Alkaline Phosphatase
Source: PLoS One. 2011 Dec 8;6(12):e28470. doi: 10.1371/journal.pone.0028470 (PMC3234256; doi:10.1371/journal.pone.0028470)
Supplement: Table S3 — Predicted residues and pairwise distances for a list of Class A, C and D β-lactamases using a Class A β-lactamase motif {Ser70, Lys73, Ser130, Lys234}. The distances are specified in the reference protein (PDB id: 2G2U) in A. For the remaining, we show the deviation from the reference value. In the Class C β-lactamases (2QZ6 for example), Ser is replaced by the stereochemically equivalent Tyr in the third position of the match. However, in some of the Class C β-lactamases (2QZ6 for example), the predicted Tyr (Tyr112) is different from the one known to be responsible for catalysis (Tyr150). The inherent shortcoming of any method that uses RSMD is its inability to distinguish between two mirror image configurations. This is evident for the Class A β-lactamase 3DW0 and the Class D β-lactamase 2HP5, which is a mirror image configuration with respect to other motifs in this set. It can also be seen that the Class A proteins match better than the Class C and Class D proteins, since we are using a Class A β-lactamase motif. One Class C β-lactamase (PDB id: 3GQZ) is an exception, possibly because this protein has had conformational changes due to fragment binding. (PDF) [file pone.0028470.s011.pdf]

Supplementary Table. 3: Predicted residues and pairwise distances for a list of Class A, C and D β-lactamases using a Class A β-lactamase motif {Ser70, Lys73, Ser130, Lys234}. The distances are specified in the reference protein (PDB id: 2G2U) in Å. For the remaining, we show the deviation from the reference value. In the Class C β-lactamases (2QZ6 for example), Ser is replaced by the stereochemically equivalent Tyr in the third position of the match. However, in some of the Class C β-lactamases (2QZ6 for example), the predicted Tyr (Tyr112) is different from the one known to be responsible for catalysis (Tyr150). The inherent shortcoming of any method that uses RSMD is its inability to distinguish between two mirror image configurations. This is evident for the Class A β-lactamase 3DW0 and the Class D β-lactamase 2HP5, which is a mirror image configuration with respect to other motifs in this set. It can also be seen that the Class A proteins match better than the Class C and Class D proteins, since we are using a Class A β-lactamase motif. One Class C β-lactamase (PDB id: 3GQZ) is an exception, possibly because this protein has had conformational changes due to fragment binding.

PDB ID	Ser70(a)	Lys73(b)	Ser130(c)	Lys234(d)	ab	ac	ad	bc	bd	cd	Score
2G2U(A)	70	73	130	234	2.7	3.5	4.4	4.2	5.3	2.6	0
1JTG(A)	70	73	130	234	0.1	-0.4	-0.2	0.0	-0.1	0.1	0.7
1O7E(A)	70	73	130	234	0.0	0.5	-0.1	1.1	0.0	-0.3	1.2
4BLM(A)	70	73	130	234	-0.0	0.2	-0.2	1.1	0.1	-0.4	1.2
3DW0(A)	130	234	70	73	0.0	-0.7	-0.4	-0.8	-0.6	-0.1	1.4
1DY6(A)	70	73	130	234	-0.0	-0.1	-1.0	1.2	-0.1	-0.5	1.6
1BUL(A)	70	73	130	234	-0.2	0.3	-0.5	1.0	-0.2	-0.4	2.0
3GQZ(C)	64	67	150	315	-0.2	0.6	-0.1	1.2	0.1	-0.3	1.7
2QZ6(C)	64	67	112	315	0.3	-2.8	-0.5	-0.4	-0.3	-2.4	5.0
1GA0(C)	64	67	150	318	-0.4	0.5	-0.2	1.5	-0.1	-0.5	3.0
2WZZ(C)	90	93	138	342	-0.7	-3.4	-0.1	-0.0	0.3	-2.6	6.5
2ZC7(C)	64	67	150	315	-0.5	0.3	-0.4	1.3	-0.1	-0.3	3.2
1FOF(C)	67	70	115	205	-0.2	0.6	-0.3	1.2	0.1	-0.9	2.4
2HP5(C)	115	205	67	70	-0.5	0.7	0.7	-0.0	0.4	-1.0	3.4
