# Supplementary material for: Active Site Detection by Spatial Conformity and Electrostatic Analysis—Unravelling a Proteolytic Function in Shrimp Alkaline Phosphatase
Source: PLoS One. 2011 Dec 8;6(12):e28470. doi: 10.1371/journal.pone.0028470 (PMC3234256; doi:10.1371/journal.pone.0028470)
Supplement: Table S4 — Pairwise distances and potential differences between pairs of residues in the reference motif from the demaminase (PDB id: 1CD5) (Residues listed in CSA database: {His143,Glu148,Asp141,Asp72}) and the unknown protein Tm0936 from Thermotoga maritima (PDB id: 1P1M) (CLASP predicted residues: {His55,Glu203,Asp279,Asp113}). (PDF) [file pone.0028470.s012.pdf]

Supplementary Table. 4: Pairwise distances and potential differences between pairs of residues in the reference motif from the demaminase (PDB id: 1CD5) (Residues listed in CSA database: {His143,Glu148,Asp141,Asp72}) and the unknown protein Tm0936 from *Thermotoga maritima* (PDB id: 1P1M) (CLASP predicted residues: {His55,Glu203,Asp279,Asp113}).

Pairwise Distances						
1P1M	8.3	4.7	7.9	6.6	14.5	8.8
1CD5	6.4	2.6	11.7	8.1	12.3	14.2
Pairwise Potential Differences						
1P1M	148.3	196.3	217.7	48.0	69.5	21.4
1CD5	141.8	152.0	263.7	10.2	121.9	111.7
