# Supplementary material for: Active Site Detection by Spatial Conformity and Electrostatic Analysis—Unravelling a Proteolytic Function in Shrimp Alkaline Phosphatase
Source: PLoS One. 2011 Dec 8;6(12):e28470. doi: 10.1371/journal.pone.0028470 (PMC3234256; doi:10.1371/journal.pone.0028470)
Supplement: Table S5 — Identity/Similarity among all Aps. (PDF) [file pone.0028470.s013.pdf]

Supplementary Table. 5: Potential difference between Lys73 and Glu166 for a motif from a Class A β-lactamase which now includes the Glu166 for a list of Class A β-lactamase proteins {Ser70, Lys73, Ser130, Lys234, Glu166}, the high potential differences observed are consistent with the theory that Lys73 is protonated in the initial stages, and acts as the general base to Ser70 only after transferring a proton to the Glu166

PDB id:	2G2U	3BFG	1O7E	1HTZ	4BLM	2QPN
K73/E166	435.4	561.2	441.5	447.3	443.5	362.4

.
