# Supplementary material for: Active Site Detection by Spatial Conformity and Electrostatic Analysis—Unravelling a Proteolytic Function in Shrimp Alkaline Phosphatase
Source: PLoS One. 2011 Dec 8;6(12):e28470. doi: 10.1371/journal.pone.0028470 (PMC3234256; doi:10.1371/journal.pone.0028470)
Supplement: Table S6 — Potential difference between Lys73 and Glu166 for a motif from a Class A β-lactamase which now includes the Glu166 for a list of Class A β-lactamase proteins {Ser70, Lys73, Ser130, Lys234, Glu166}, the high potential differences observed are consistent with the theory that Lys73 is protonated in the initial stages, and acts as the general base to Ser70 only after transferring a proton to the Glu166. (PDF) [file pone.0028470.s014.pdf]

Supplementary Table. 6: Identity/Similarity among all APs

	1ED9 E. coli	1K7H Shrimp	2IUC Antarctic Bacterium	1EW2 human placenta	2X98 Halobacterium salinarum	3E2D Vibrio strain G15-2
1ED9	100/100	26.5/38.1	26.4/40.6	26.5/40.1	27.4/38.7	22.0/32.0
1K7H	-	100/100	23.5/35.6	37.2/51.4	25.8/37.5	25.0/37.4
2IUC	-	-	100/100	21.4/34.8	23.4/37.4	23.8/35.1
1EW2	-	-	-	100/100	25.9/36.4	23.7/35.2
2X98	-	-	-	-	100/100	25.0/36.7
3E2D	-	-	-	-	-	100/100
