# Supplementary material for: Active Site Detection by Spatial Conformity and Electrostatic Analysis—Unravelling a Proteolytic Function in Shrimp Alkaline Phosphatase
Source: PLoS One. 2011 Dec 8;6(12):e28470. doi: 10.1371/journal.pone.0028470 (PMC3234256; doi:10.1371/journal.pone.0028470)
Supplement: Table S7 — Dataset. (PDF) [file pone.0028470.s015.pdf]

Supplementary Table. 7: **Dataset**

File Name	Description	Number
list.1	All proteins in the PDB Database more than 100 amino acids long	47940
list.2	Class A, C and D β-lactamases having 80% or less similarity	15
list.3	non-redundant proteins from http://www.ncbi.nlm.nih.gov/Structure/VAST/nrpdb.html	3000
list.4	Serine proteases of the class trypsin having 80% or less similarity	7
list.5	Random 10 proteins from the PDB	10
list.6	Proteins with motifs extracted from CSA Database	391
list.7	Random set of proteins annotated as putative in the PDB database	52
list.8	Serine proteases of the class subtilisin having 80% or less similarity	7
list.9	Best matches obtained using a 3D congruence check	
	with a serine protease motif having 80% or less similarity	1695
